# A practical ‘How-To’ Guide to plain language summaries (PLS) of peer-reviewed scientific publications: results of a multi-stakeholder initiative utilizing co-creation methodology

**DOI:** 10.1186/s40900-022-00358-6

**Published:** 2022-06-02

**Authors:** Laura Dormer, Thomas Schindler, Lauri Arnstein Williams, Dawn Lobban, Sheila Khawaja, Amanda Hunn, Daniela Luzuriaga Ubilla, Ify Sargeant, Anne-Marie Hamoir

**Affiliations:** 1 Future Science Group, London, UK; 2 Boehringer Ingelheim Pharma GmbH & Co. KG, Biberach a. d. Riss, Germany; 3 Envision Pharma Group, London, UK; 4 Envision Pharma Group, Wilmslow, UK; 5 World Alliance of Pituitary Organizations, Zeeland, The Netherlands; 6 A J Hunn Associates, London, UK; 7 Patient Focused Medicines Development, The Synergist, Brussels, Belgium; 8 Twist Medical, Burlingame, CA USA

**Keywords:** Plain language summary, Patient engagement, Scientific publication

## Abstract

**Background:**

Peer-reviewed scientific publications and congress abstracts are typically written by scientists for specialist audiences; however, patients and other non-specialists are understandably interested in the potential implications of research and what they may mean for them. Plain language summaries (PLS)—summaries of scientific articles in easy-to-read language—are emerging as a valuable addition to traditional scientific publications. Co-creation of PLS with the intended audience is key to ensuring a successful outcome, but practical guidance on how to achieve this has been lacking.

**Methods:**

Building on the Patient Engagement (PE) Quality Guidance previously developed by Patient Focused Medicines Development (PFMD), a multi-stakeholder working group (WG) of individuals with patient engagement experience and/or expertise in PLS was established to develop further activity-specific guidance. PLS guidance was developed through a stepwise approach that included several rounds of co-creation, public consultation (two rounds), internal review and a final external review. The iterative development process incorporated input from a wide variety of stakeholders (patient representatives, industry members, publishers, researchers, medical communications agencies, and public officials involved in research bodies). Feedback from each step was consolidated by the WG and used for refining the draft guidance. The final draft was then validated through external consultation.

**Results:**

The WG comprised 14 stakeholders with relevant experience in PE and/or PLS. The WG developed a set of 15 ethical principles for PLS development. These include the necessity for objective reporting and the absence of any promotional intent, the need for balanced presentation, the importance of audience focus, the need to apply health literacy principles, and the importance of using inclusive and respectful language. The first public consultation yielded 29 responses comprising 478 comments or edits in the shared draft guidance. The second public consultation was an online survey of 14 questions which had 32 respondents. The final ‘How-To’ Guide reflects feedback received and provides a rational, stepwise breakdown of the development of PLS.

**Conclusions:**

The resulting ‘How-To’ Guide is a standalone, practical, ready-to-use tool to support multi-stakeholder co-creation of PLS.

**Supplementary Information:**

The online version contains supplementary material available at 10.1186/s40900-022-00358-6.

## Plain English summary

We wanted to create practical guidance for people who are interested in developing plain language summaries of publications (PLS for short). PLS are summaries of scientific research published in journals or presented at conferences and are written in language that is easy to read and understand. We focused on how to involve patients in developing PLS, as they are often an important audience for these summaries. We brought together a group of people who had experience in PLS and patient involvement. As a working group, we wrote the first version of the ‘How-To’ Guide. Then we asked for feedback from others experienced in patient involvement and also from members of the general public. We got feedback on how we could improve what was in the guidance and also on how useful and user-friendly the guidance was. We used this feedback to create the final version of the ‘How-To’ Guide which is freely available online from https://pemsuite.org/How-to-Guides/WG5.pdf.

**Supplementary Information:**

The online version contains supplementary material available at 10.1186/s40900-022-00358-6.

## Introduction

Peer-reviewed scientific publications and congress abstracts are the established channels through which researchers share data with their peers and are typically written by scientists for this specialist audience. The technical language used, and complex data included, are often impenetrable for non-specialist audiences (i.e., those who are not experts in the subject matter). Non-specialists include patients and their caregivers who are understandably interested in the potential implications of research and want to find out what it may mean for them and the conditions they are living with [1].

Plain language summaries (PLS) are summaries of scientific articles and congress abstracts written in easy-to-read, non-technical language [2]. They are emerging as a valuable addition to scientific publications because they have the potential to increase the understanding of scientific data, by making complex information more accessible to wider audiences than they would otherwise reach [3]. This includes healthcare professionals from different fields, patients, patient organizations, caregivers, and the general public [4]. PLS may also facilitate patient–healthcare professional communication by improving knowledge and understanding, which, in turn, could contribute to shared decision making [1]. Importantly, PLS are only of value when facts, numbers, and conclusions are conveyed truthfully and objectively, without promotional intent or spin [5]. PLS can only fulfil their objectives if readers can fully trust that all relevant data—including information on the uncertainty of research conclusions—have been made available to them. Thus, PLS need to operate in an ethical space.

Although the number of PLS associated with peer-reviewed publications is still relatively low, it is increasing [5]. However, there is currently wide variation in content, format, quality, and location (i.e., where people can access them) of PLS [6]. Studies are ongoing with the aim of providing clear guidance, and minimum standards for PLS have recently been proposed [7–9].

Co-creation of PLS, i.e., with collaboration between researchers and the intended audience, is key to ensuring a successful outcome. However, in current practice, patient involvement is often restricted to the late stages of PLS development, for example the review process. The need for practical ‘How-To’ guidance that will help to ensure patient involvement early on was recognized by Patient Focused Medicines Development (PFMD) [10]. PFMD is a collaboration of health stakeholders, including publishers, patient organizations and pharmaceutical companies, whose aim is to facilitate patient engagement across the medicines development lifecycle.

Here we describe the development of a ‘How-To’ Guide for multistakeholder co-creation of PLS for peer-reviewed publications. The iterative development process incorporated input from a wide variety of stakeholders (patient representatives, industry members, publishers, researchers, medical communications agencies, and public officials involved in research bodies) to produce an actionable guidance document with practical advice and examples. A PLS of this publication describing the development of the ‘How-To’ Guide is available within the Additional file 1.

## Methods

**Fig. 1:**
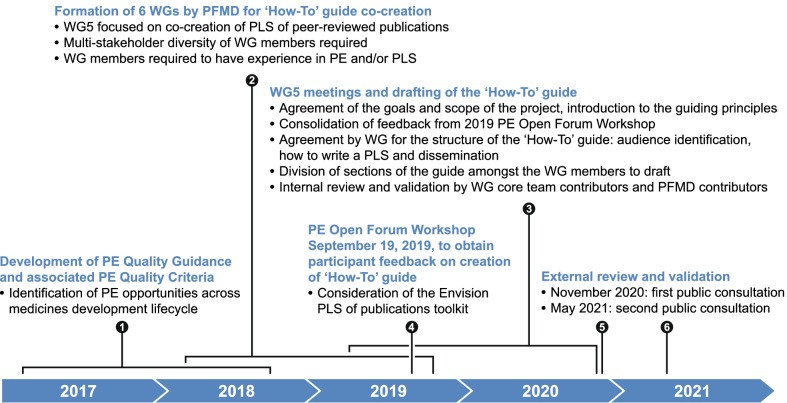
Milestones along the PLS co-creation process [11, 14, 15]. PE, patient engagement; PFMD, Patient Focused Medicines Development; WG, working group

The methods used for co-creation of the ‘How-To’ Guide have been previously described in detail [11–13]. Briefly, a landscape analysis and a public consultation were carried out and the feedback was used to develop the Patient Engagement Quality Guidance (PEQG). The feedback also helped to identify priority activities for patient engagement (PE) in medicines development and lifecycle. ‘How-To’ Guides are then co-produced through several workshops and in multi-stakeholder working groups (WGs) [11]. WG5 was established to focus on the development of a ‘How-To’ Guide for PLS co-development. The stepwise approach included several rounds of co-creation, public consultation, internal review, and final external review. Feedback from each step in the review process was consolidated, divided into themes, and prioritized based on the proportion or amount of feedback per theme and WG assessment of its relative importance. This feedback was used to refine the draft ‘How-To’ Guide, which was then validated through additional consultation [13]. Specific milestones along the PLS co-creation process are shown in Fig. 1.

A completed Guidelines for Reporting Involvement of Patients and the Public (GRIPP2) Short Form [16] is included as Additional file 2.

WG5 members were required to have PE experience and/or expertise in PLS. WG5 core team contributors were defined as those with active involvement in most aspects of the ‘How-To’ Guide co-creation, from conceptualization to finalization. Contributors from PFMD provided substantial feedback at the draft stages of the ‘How-To’ Guide.

## Results

### WG5 core team contributors and PFMD contributors

**Fig. 2:**
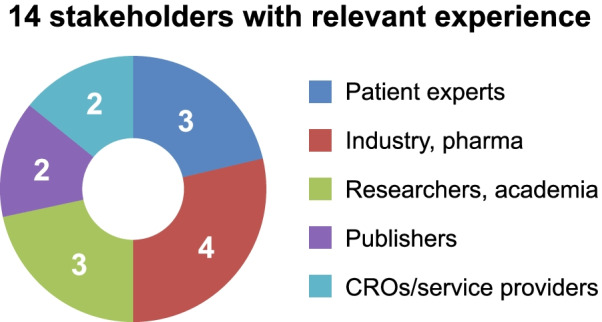
Stakeholder diversity of WG5 contributors. CRO, contract research organizations; WG, working group

WG5 core team contributors consisted of 14 stakeholders with relevant experience in PE and/or PLS (Fig. 2). Details of the WG5 core team contributors as well as the PFMD contributors can be found in Additional file 3: Table S1.

### Public consultation

**Fig. 3:**
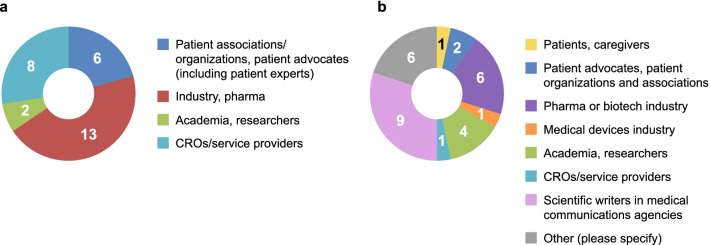
Stakeholder groups providing feedback in the first (**a**) and second (**b**) public consultation. CRO, contract research organizations

The draft ‘How-To’ Guide underwent two rounds of public consultation. In the first round, reviewers commented on the content for improvement, and in the second round, feedback was gathered on the usability of the ‘How-To’ Guide and the associated user experience. Responses from both consultations were used to refine the draft ‘How-To’ Guide. The process included revision of the executive summary of the Guide, addition of enhanced visual elements, improved alignment with PEQG [11], addition of an appendix with links to relevant tools and resources, and addition of a glossary of abbreviations. Results of the public consultations are summarized in Table 1 (the full consultation results are provided in Additional file 4: Table S2). Stakeholder groups providing feedback in each public consultation are listed in Fig. 3.

**Table 1:** Summary of public consultations

	First public consultation	Second public consultation
Dates	September to November 2020	February to April 2021
Purpose	Seek suggestions for content improvement	Assess usability and user experience
Shared with	35 PFMD members 95 contributors across 7 WGs	General public
Format	Google docs	Online survey with 14 questions
Responses	29 respondents representing 16 organizations	30 respondents to the survey + 2 respondents over email (1 external consultant in PE, 1 industry)
Feedback received	478 comments/edits	32 responses
Outcomes	Executive summary Visual enhancements Alignment with PEQG Appendix of tools/resources Glossary of abbreviations	Addition of more resources Final minor modifications Confirmation of usability

*PE* Patient Engagement, *PEQG* Patient Engagement Quality Guidance, *PFMD* Patient Focused Medicines Development, *WG *Working Group

**Fig. 4:**
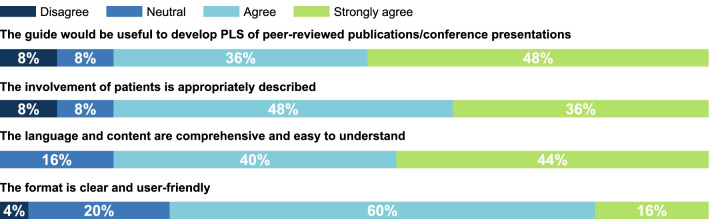
Overall impressions of the draft ‘How-To’ Guide from the second public consultation (n = 25). PLS, plain language summary

Results from the second public consultation indicated that 60% of respondents would use the Guide to educate people in their network or organization on the development of a PLS, 48% would use it to identify gaps and opportunities for PE, and 40% would use it to help them improve the quality and consistency of PE activities they are involved in. In addition, 71% of respondents indicated that they would use the ‘How-To’ Guide as part of their role or in their work and 60% said they would like to receive additional support for implementing the recommendations in the ‘How-To’ Guide, including examples and best practice, guidance on measuring impact, and implementation training. Respondents’ overall impressions of the ‘How-To’ Guide were also captured during the second public consultation (Fig. 4).

### ‘How-To’ Guide for PLS co-creation: content

WG5 developed a set of 15 ethical principles that are important for developing trustworthy and helpful PLS. The principles cover the necessity for objective reporting, the need to apply health literacy principles, the importance of audience focus and the absence of any promotional intent, the need for balanced presentation, and the importance of using inclusive and respectful language; the principles also cover practical aspects. These principles are the foundations of any PLS. After firmly anchoring PLS writing in a set of ethical principles, the ‘How-To’ Guide proceeds to a stepwise breakdown of the development of a PLS.

#### Step 1: Rationale and scope of your PLS

It is important to develop a rationale for writing a PLS. Before the writing takes place, there must be clarity around why a certain scientific publication has been selected for development into a PLS. It is particularly important to have a transparent process across an organization for the development of PLS, to avoid publication bias by selectively providing PLS for studies that have favorable results. Reasons for developing a PLS could include the novelty of the data, availability of phase 3 clinical trial data, studies with particularly patient-relevant outcomes, the uniqueness of the scientific approach, or the needs of a certain audience. At the outset, it is important to consider the resource needs, the target journal, and the accessibility of the PLS (i.e., where it will be made available). As co-creation is a key aspect of PLS development, an evaluation of the available infrastructure, the extent of outreach into the target community and the associated organizational and financial resources should be carried out.

#### Step 2: Identify your target audience

To maximize the value of a PLS, the target audience should be clearly defined before the start of the writing process. The identified target audience will critically influence the resource needs in the co-creation process and will determine the administrative and operational complexity of the PLS writing. For example, once patients with a particular condition are chosen to be PLS co-creators, contact groups in the disease area should also be identified, and the appropriate contractual and financial agreements developed, before the PLS content is planned in more detail.

#### Step 3: Consider the dissemination channels for the PLS

Before any detailed planning of the content selection and the writing, it is essential to consider the dissemination of the PLS based on the identified target audience. The available options need to be evaluated as this will determine the amount of aggregation and summarization of the data from the original article. For example, if a manuscript and its PLS are to appear in the same issue of a journal, certain details may be omitted from the PLS and provided solely as references. Furthermore, some journals may allow the inclusion of additional file that could be used for additional infographics. Free access to a PLS is paramount, and this needs to be kept in mind when choosing a journal that will adequately serve the needs of the target audience.

#### Step 4: Identify your key stakeholders for co-creation of PLS

Before a PLS is written, it is important to identify the key stakeholders to engage with for co-creation. Step 2 discusses identifying the target audience for the PLS, and this is a good starting point to finding the best stakeholders to ensure the finished PLS is understandable and relevant. A broad range of stakeholders, including patients, is desirable, to help with the various activities for developing a successful PLS (e.g., selection of the publication to summarize; reviewing and providing feedback on drafts of the PLS). The PLS co-creators should determine whether they have the appropriate reach into the target audience, or whether it would be better to establish new co-creation relationships. Resourcing (e.g., contracting, payment, technical infrastructure) and, most importantly, any applicable legal requirements need to be considered, to ensure those relationships can be maintained throughout the PLS preparation.

#### Step 5: Write your PLS

Only when steps 1–4 have been followed and the PLS team has been assembled, should writing begin. For this stage, the PLS co-creators should establish an appropriate infrastructure, and participants should agree what their roles are and how they will interact. Everyone should make sure they are familiar with the ethical principles for PLS co-creation (more information is provided on this in the ‘Ethical Considerations for PLS’ section in the ‘How-To’ Guide [17]).

The aim of a PLS is to provide a clear, accessible summary of the content of a scientific publication for non-specialist readers. Based on the target audience, the PLS co-creators should decide the appropriate reading age and literacy level of the PLS, and how it will be structured (including any visual or audio formats that might be useful). Information should be presented visually wherever possible (e.g., in the form of diagrams and infographics), and time should be taken to consider how this can be done effectively.

Review by target audience members can be used to ensure the quality of the PLS, and to guide revisions accordingly. Although online readability tools may also help, they cannot be a substitute for target audience feedback. Writing in plain language is challenging, particularly for those professionally accustomed to technical/scientific terminology. This part of the review process is vital for ensuring that the PLS content is appropriate. It is also important to follow the requirements/guidelines provided by the journal to which the PLS will be submitted.

#### Step 6: Disseminate your PLS

Once the PLS is published, it may be shared in a variety of ways, such as in print, in online repositories, or on relevant websites; the best channels to use should be chosen based on the target audience. In whatever format the PLS is shared, it must reference the scientific manuscript it is summarizing, or contain links to it. The use of social media for dissemination depends on the legal restrictions in some countries and the corresponding compliance rules in large organizations.

Several entities might share the PLS, including the publishing journal, the PLS co-creators and their institutions, healthcare providers, patients, advocates, and medical societies. For this to happen, though, all institutional policies and the requirements of the relevant country-level regulatory entities must be adhered to. Copyright policies of the publishing journal and the journal where the PLS is being submitted (if different) should also be considered, because they may limit the dissemination of the PLS.

#### Step 7: Track dissemination and measure success

As a final step, ways to monitor the impact and value of the PLS should be developed, as all feedback is useful for informing improvements of the process for future efforts. Various metrics may be available to help measure success, depending on where a PLS is located/hosted. The journal site/website/repository that hosts the PLS might provide metrics, such as the number of times it has been viewed or downloaded, or the attention it has received online, e.g., on social media or other commentaries. Posts on social media that link to the PLS may be liked or shared and monitoring this activity can provide an indication of the reach of the PLS among the target audience.

More importantly, success of dissemination routes should also be measured in other ways, such as whether the PLS is shared by patient organizations and healthcare providers. This is essential in determining to what extent a target audience was reached that may not be able to access PLS through traditional academic channels (e.g., via peer-reviewed journals or PubMed).

## Discussion

The ‘How-To’ Guide has been developed using an iterative and robust methodology [11–13] to meet an identified need for a practical, ready-to-use tool for multi-stakeholder co-production of PLS. The methodology adhered to key principles of co-creation [11] and included internal reviews (through WG5 workshops) and external review (at the Patient Engagement Open Forum workshop in September 2019; Fig. 1), as well as two rounds of public consultation. All feedback was used to refine the guidance at each step. A key learning experience has been the importance of providing a Guide that is easy to understand in a format that is user-friendly [11].

In their early discussions, the multi-stakeholder WG widely agreed that the PE guidance tool should be accessible and support diverse audiences with varying levels of experience in PE. Accordingly, the ‘How-To’ Guide for PLS has been designed for use without the need for additional guidance: the language and tone are clear and understandable, the structure is self-explanatory, and the layout is intuitive and easy to navigate. Responses from the second public consultation confirmed that the ‘How-To’ Guide is useful for development of PLS, describes the involvement of patients appropriately, has language and content that is comprehensive and easy to understand, and is displayed in an easy-to-use format. Selected resources for PLS development (identified as helpful by WG contributors), complementary tools, and good-practice examples are available directly from the ‘How-To’ Guide. In practice this means that anyone looking to be a co-creator of a PLS of a peer-reviewed publication can use the Guide without additional support. An executive summary of the ‘How-To’ Guide and its annexes with links to additional resources are in the Additional file 5.

One learning point from this project was that during the consultation rounds (including public consultation and live feedback sessions), there were challenges in mobilizing participants on the topic of PLS. For example, during the public consultation, although it was promoted via social media as well as through WG contributor and PFMD networks, the response rate was relatively low (32 respondents). Reasons for this may be the novelty of the format, the public not being used to this type of consultation, or a lack of confidence or experience to review and comment. However, quality of the feedback that was received highlighted that those who did provide input had a good level of understanding of the topic. Nevertheless, the low response rate indicates that there is considerable need for more effective communication on the importance of PLS, and to encourage more stakeholders to participate in such activities.

Although first instigated in 2010 by the *British Journal of Dermatology*, there are currently few journals that routinely provide the opportunity for PLS [6, 18]. Communication campaigns targeting patient advocacy groups, selected presentations during PE conferences with broader audiences, and introduction webinars explaining PLS principles are potential approaches for addressing current barriers and providing support for PLS implementation.

For those audiences who are likely to be aware of PLS already (e.g., medical writers, communication professionals, editors/publishers), we aim to maximize dissemination of the ‘How-To’ Guide through their connections and networks. In order to reach priority stakeholders in the medicines development continuum (e.g., patient advocacy/organizations and industry), the ‘How-To’ Guide [17] has been uploaded onto the PFMD PE Management Suite [19], a central repository that allows open access to all PFMD tools. This guidance was developed for peer-reviewed scientific publications in the field of medicine and pharmaceutical development. However, the principles can easily be applied to subject matters beyond pharmaceuticals. They are relevant for all peer-reviewed scientific publications where there is a need to work with non-specialists to co-create a PLS.

The ‘How-To’ Guide should be perceived as a working and organic document that will need to be revisited and updated as the PLS landscape evolves and matures. While we understand and have established the value of PLS, maintaining the quality of the Guide will be important to ensure its contents remain relevant. For example, if a PLS is to be translated, the translation would need to be accurate and there would ideally be back translation, as well as patient input, to ensure the PLS retains its original meaning and remains a patient-friendly document. It is also essential for both sponsors and journals to have a consistent policy for the development and publishing of PLS. This means there should be transparent, prospective, and objective selection criteria for choosing publications from which to develop PLS, and for deciding how and when they will be published in order to prevent publication bias. For example, one criterion from a sponsor could be a commitment to producing PLS for all phase 3 trials, regardless of outcomes. PLS on single trials should also include a disclaimer on the limitations and generalizability of reports from such studies.

To further measure the usability and impact of the ‘How-To’ Guide among stakeholder groups, PFMD is seeking organizations that will volunteer to pilot its use and provide any insights and learnings to our WG. PFMD also welcomes feedback on this tool via pfmd@thesynergist.org and hopes that the Guide will facilitate consistent multi-stakeholder PLS co-production.

## Supplementary Information

**Additional file 1:** Publication PLS.

**Additional file 2:** GRIPP2 Short Form.

**Additional file 3: Table S1.** WG5 core team contributors and PFMD network contributors.

**Additional file 4: Table S2.** Public consultation survey results.

**Additional file 5:** ‘How-To’ Guide: executive summary and annexes.

## Data Availability

The datasets supporting the conclusions of this article are included within the article (and its additional files).

## Abbreviations

CRO: Contract research organizations
PE: Patient engagement
PEQG: Patient Engagement Quality Guidance
PFMD: Patient Focused Medicines Development
PLS: Plain language summaries
WG: Working group
