# Supplementary material for: A practical ‘How-To’ Guide to plain language summaries (PLS) of peer-reviewed scientific publications: results of a multi-stakeholder initiative utilizing co-creation methodology
Source: Res Involv Engagem. 2022 Jun 2;8:23. doi: 10.1186/s40900-022-00358-6 (PMC9164486; doi:10.1186/s40900-022-00358-6)
Supplement: Supplementary file 1 — Additional file 1: Publication PLS. [file 40900_2022_358_MOESM1_ESM.pdf]

A ‘How-To’ guide for plain language summaries of publications

Date of summary: November 2021

The purpose of this plain language summary is to help you understand how we developed a guidance document.

1 What did this project look at?

- We wanted to create guidance for people who are interested in developing plain language summaries of publications (PLS for short).

PLS are summaries of scientific research published in journals or presented at conferences (called peer-reviewed publications).

They are written using language that is easy to read and understand.

We wanted this document to be a practical ‘How-To’ guide.

We focused on how to involve patients in developing PLS, as they are an important audience for these summaries.

2 How was the ‘How-To’ guide created?

- Patient Focused Medicines Development (PFMD for short) brought together a working group of people with experience in PLS and patient involvement.
- This working group had 14 members:

4 people working in the pharmaceutical industry

3 patients

3 people doing research in a university or nonprofit organization

2 journal publishers

2 people providing services to the pharmaceutical industry

- As a working group, we wrote the first version of the ‘How-To’ guide.
  – We used the principles from PFMD’s Patient Engagement Quality Guidance. This describes how best to work with patients on different types of projects.

- Then we looked for feedback using 2 rounds of review:

Round 1: September–November 2020

We asked for feedback from:

- Members of PFMD
- Members of other PFMD working groups looking at patient involvement in medicines development

We asked people to comment on the guide online

29
people responded

They gave us **478 comments and edits** about how we could **improve the content** of the guidance.

Round 2: February–April 2021

We asked for feedback from:

Members of the general public

We used a online survey

32
people responded

They gave us feedback about how **useful and user-friendly** the guidance was.

- We used this feedback to create the final version of the 'How-To' guide.

3 What does the 'How-To' guide contain?

The 'How-To' guide contains:

15 ethical principles to help make sure that PLS are balanced, relevant, and accessible for their target audiences.

7 steps people can follow to develop a PLS.

1. Decide the reasons why you are doing a PLS, and agree on the scope of the PLS
2. Identify your target audience
3. Consider how and where you will share the PLS
4. Identify who you will work with to co-create the PLS
5. Write your PLS
6. Publish and share your PLS
7. Track your PLS and measure success

4 What were the main conclusions reported by the working group?

- Limited guidance is available at the moment to help people who are interested in developing PLS of peer-reviewed publications.
- We created a practical ‘How-To’ guide for PLS, based on the principle that PLS should be co-created with the target audience.
- Raising awareness of PLS is a priority.
  - A limitation of our project is the relatively low numbers of people who responded during the rounds of review.
  - We are committed to sharing information on PLS as widely as possible, including this guidance.

5 Are there any plans for further projects?

- We plan to continue working on the ‘How-To’ guide, to make sure it stays up-to-date and relevant.
- We are interested in receiving further feedback and hearing from any groups who would like to use and test the ‘How-To’ guide.
  - Please contact pfmd@thesynergist.org

6 Who sponsored this study?

PFMD sponsored the development of the ‘How-To’ guide, with the active, voluntary participation of the working group members.

Further information

- The ‘How-To’ guide is available online and is free to access here: https://pemsuite.org/How-to-Guides/WG5.pdf
- You can find other ‘How-To’ guides and the Patient Engagement Quality Guidance at PFMD’s Patient Engagement Management suite: https://pemsuite.org/

The full title of this article is: Plain language summaries of peer-reviewed publications and conference presentations: Practical ‘How-To’ guide for multi-stakeholder co-creation. You can find the full article here: <insert link once available>

You can access the full article for free.

Summary prepared by Dr Lauri Arnstein Williams, MA MBBS, Envision Pharma Group. This summary was reviewed by patient champion Antony Chuter. The other co-authors of the full article were involved in preparing this summary.
