# Supplementary material for: A practical ‘How-To’ Guide to plain language summaries (PLS) of peer-reviewed scientific publications: results of a multi-stakeholder initiative utilizing co-creation methodology
Source: Res Involv Engagem. 2022 Jun 2;8:23. doi: 10.1186/s40900-022-00358-6 (PMC9164486; doi:10.1186/s40900-022-00358-6)
Supplement: Supplementary file 2 — Additional file 2: GRIPP2 Short Form. [file 40900_2022_358_MOESM2_ESM.docx]

**GRIPP2 short form**

| **Section and topic** | **Item** | **Reported on page no.** |
| --- | --- | --- |
| 1: Aim | Report the aim of PPI in the study | 4 and 5 |
| 2: Methods | Provide a clear description of the methods used for PPI in the study | 5 and 6 |
| 3: Study results | Outcomes―Report the results of PPI in the study, including both positive and negative outcomes | 6, 7 and 8 |
| 4: Discussion and conclusions | Outcomes―Comment on the extent to which PPI influenced the study overall. Describe positive and negative effects | 13 and 14 |
| 5: Reflections/critical perspective | Comment critically on the study, reflecting on the things that went well and those that did not, so others can learn from this experience | 13 and 14 |

PPI, patient and public involvement.
