# Supplementary material for: A practical ‘How-To’ Guide to plain language summaries (PLS) of peer-reviewed scientific publications: results of a multi-stakeholder initiative utilizing co-creation methodology
Source: Res Involv Engagem. 2022 Jun 2;8:23. doi: 10.1186/s40900-022-00358-6 (PMC9164486; doi:10.1186/s40900-022-00358-6)
Supplement: Supplementary file 3 — Additional file 3: Table S1. WG5 core team contributors and PFMD network contributors. [file 40900_2022_358_MOESM3_ESM.docx]

**Supplementary Table 1** WG5 core team contributors and PFMD network contributors

| **WG5 core team contributors** |
| --- |
| **Name Affiliation^a^**  Antony Chuter Patient champion  Avishek Pal Novartis  Begonya Nafria Escalera San Juan de Deu Children’s Hospital  Dawn Lobban Envision Pharma  Elena Conroy Sage Publishing  Jennifer Preston eYPAGNet, University of Liverpool  Laura Dormer Future Science Group  Laurence Rouxhet GlaxoSmithKline  Lauri Arnstein Williams Envision Pharma  Lauryn Uribe Gilead  Paula Wray University of Oxford  Sheila Khawaja World Alliance of Pituitary Organizations  Simon Stones Patient/Health Advocate and Consultant, Collaboro Consulting  Thomas Schindler Boehringer Ingelheim |
| **PFMD network contributors** |
| **Name Affiliation^a^**  Ana Sofia Correia ASC Translations (Portugal)  Anke-Peggy Holtorf Health Outcomes Strategies  Annekatrin Krause Novartis  Beyza Klein Novartis  Caragh Murray Janssen  Carole Scrafton Patient advocate/expert/Fibro Flutters  Cynthia Arnold Janssen  Deborah Collyar Patient Advocates in Research  Debra Guerreiro Janssen  Duane Sunwold Patient, Patient advocate/National Kidney Foundation  Dyan Bryson Inspired Health Strategies  Ellen Baum Janssen  Gloria Stone G. Stone Connections  Jeanette Ryan GlaxoSmithKline  Jessica Valencia Novartis  Linda Feighery UCB  Mary Murray National Minority Quality Forum  Merry Saba Novartis  Olsen Rikke Egelund Roche  Paola Kruger Patient Expert/EUPATI  Paul Farrow Oxford Pharmagenesis Ltd (UK)  Phil Posner PCORI  Sanjay Bagani Xogene Services, LLC  Sarah Griffiths Open Pharma  Severine Wollenschneider Roche  Sharlynda Horton Gilead  Simon Page Ipsen  Steph Macdonald Oxford PharmaGenesis Ltd (UK)/Open Pharma  Tiziana Fragiacomo Novartis |

^a^Affiliation correct at time of contribution. PFMD, Patient Focused Medicines Development; WG, working group.
