# Supplementary material for: A practical ‘How-To’ Guide to plain language summaries (PLS) of peer-reviewed scientific publications: results of a multi-stakeholder initiative utilizing co-creation methodology
Source: Res Involv Engagem. 2022 Jun 2;8:23. doi: 10.1186/s40900-022-00358-6 (PMC9164486; doi:10.1186/s40900-022-00358-6)
Supplement: Supplementary file 4 — Additional file 4: Table S2. Public consultation survey results. [file 40900_2022_358_MOESM4_ESM.docx]

**Supplementary Table 2**

Public consultation results

Plain language summaries (PLS) of peer-reviewed publications and conference presentations: practical ‘How-To’ Guide for multi-stakeholder co-creation

| Question 1 | | | | | | |
| --- | --- | --- | --- | --- | --- | --- |
| **What group or affiliation best describes you?** | | | | |  |  |
| Answer Choices | | | | | Responses | |
| Patients and caregivers | | | | | 3.33% | 1 |
| Patient advocates, patient organizations and associations | | | | | 6.67% | 2 |
| Healthcare professionals | | | | | 0.00% | 0 |
| Pharmaceutical companies or biotech industry | | | | | 20.00% | 6 |
| Medical Devices industry | | | | | 3.33% | 1 |
| Academia and researchers | | | | | 13.33% | 4 |
| CRO/ Service providers | | | | | 3.33% | 1 |
| Research Funders | | | | | 0.00% | 0 |
| Publishers | | | | | 0.00% | 0 |
| Policy makers | | | | | 0.00% | 0 |
| Regulators | | | | | 0.00% | 0 |
| Payers | | | | | 0.00% | 0 |
| Health Technology assessment (HTA) organizations | | | | | 0.00% | 0 |
| Scientific writers in communication agencies | | | | | 30.00% | 9 |
| Scientific/medical editors | | | | | 0.00% | 0 |
| Other (please specify) | | | | | 20.00% | 6 |
|  | | | | | **Answered** | **30** |
|  | | | | | **Skipped** | **0** |
| Question 2 | | |  |  |  |  |
| **Where are you located?** |  |  | |  |  |  |
| Answer Choices | Responses | |  |  |  |  |
| Afghanistan | 3.33% | 1 | |  |  |  |
| Australia | 3.33% | 1 | |  |  |  |
| Belgium | 3.33% | 1 | |  |  |  |
| Germany | 10.00% | 3 | |  |  |  |
| Italy | 3.33% | 1 | |  |  |  |
| Japan | 3.33% | 1 | |  |  |  |
| Netherlands | 6.67% | 2 | |  |  |  |
| South Africa | 3.33% | 1 | |  |  |  |
| Switzerland | 6.67% | 2 | |  |  |  |
| United Kingdom of Great Britain and Northern Ireland | 40.00% | 12 | |  |  |  |
| United States of America | 16.67% | 5 | |  |  |  |
|  | **Answered** | **30** | |  |  |  |
|  | **Skipped** | **0** | |  |  |  |
|  |  |  | |  |  |  |

| Question 3 | | |
| --- | --- | --- |
| **How much experience do you have in patient engagement? Please choose from the following options** | | |
| Answer Choices | Responses | |
| Advanced: I am actively part of patient engagement projects | 63.33% | 19 |
| Intermediary: I am sometimes involved in patient engagement projects | 30.00% | 9 |
| Beginner: Currently I have no previous experience, but I am planning to do patient engagement projects within the next year | 3.33% | 1 |
| None: I have no experience working on patient engagement activities but am interested in doing so | 3.33% | 1 |
|  | **Answered** | **30** |
|  | **Skipped** | **0** |

| Question 4 | | | |  |  |  |
| --- | --- | --- | --- | --- | --- | --- |
| **Please read the following statements and choose the option that represents best your opinion** | |  |  |  |  |  |
|  | Strongly disagree | Disagree | Neutral | Agree | Strongly agree | Total |
| I think this How-to Guide will be useful to develop PLS for peer-reviewed publications and conference presentations. | 0.00% | 8.00% | 8.00% | 36.00% | 48.00% | 25 |
| I think this How-to Guide will be useful to involve patients in the co-creation of my PLS. | 0.00% | 0.00% | 12.00% | 52.00% | 36.00% | 25 |
| I think the involvement of patients in the co-creation is appropriately described in this How-to Guide | 0.00% | 8.00% | 8.00% | 48.00% | 36.00% | 25 |
| The language and content used in the How-to Guide is comprehensive and easy to understand | 0.00% | 0.00% | 16.00% | 40.00% | 44.00% | 25 |
| The format of the How-to Guide is clear and user-friendly | 0.00% | 4.00% | 20.00% | 60.00% | 16.00% | 25 |
| In the context of my role or my work, this How-to Guide provides me with appropriate and practical patient engagement guidance | 0.00% | 0.00% | 24.00% | 52.00% | 24.00% | 25 |
| I think this How-to Guide will be useful for me to do meaningful patient engagement | 0.00% | 4.00% | 20.00% | 48.00% | 28.00% | 25 |
|  |  |  |  |  | **Answered** | **25** |
|  |  |  |  |  | **Skipped** | **5** |

| Question 5 | | | | |
| --- | --- | --- | --- | --- |
| **Continue the following sentence with options that fit best in your opinion: “I can use this How-to guide to...** | | | | |
| Answer Choices | | Responses | | |
| Improve the quality and consistency of patient engagement activities I am involved in | | 40.00% | | 10 |
| Better plan and develop patient engagement activities in PLS | | 32.00% | | 8 |
| Identify gaps and opportunities for patient engagement | | 48.00% | | 12 |
| Educate people in my network /organisation about patient engagement in the development of a PLS | | 60.00% | | 15 |
| Not applicable for me | | 8.00% | | 2 |
| Other (please specify) | | 12.00% | | 3 |
|  | | **Answered** | | **25** |
|  | | **Skipped** | | **5** |
| Question 6 | | |  |  |
| **Would you need support in implementing the recommendations in this How-to Guide?** | | |  |  |
| Answer Choices | Responses | |  |  |
| No | 60.00% | 15 |  |  |
| Yes | 40.00% | 10 |  |  |
|  | **Answered** | **25** |  |  |
|  | **Skipped** | **5** |  |  |

| Question 7 | | |
| --- | --- | --- |
| **Please specify the kind of support you might need** |  |  |
| Answer Choices | Responses | |
| I’d like to see examples and best practices how others have put this guide into action | 88.89% | 8 |
| Training showing me how to put it in action | 44.44% | 4 |
| Support from my organization to apply it in practice | 44.44% | 4 |
| Support in measuring impact when putting it into action | 77.78% | 7 |
| I would need more resources or help to identify more resources (please use the text box to clarify which resources) | 11.11% | 1 |
| Other (please specify) | 22.22% | 2 |
|  | **Answered** | **9** |
|  | **Skipped** | **21** |

| Question 8 | | |
| --- | --- | --- |
| **In the context of your role or your work, would you use this guide?** | | |
| Answer Choices | Responses | |
| Yes | 70.83% | 17 |
| No (Please specify why not) | 29.17% | 7 |
|  | **Answered** | **24** |
|  | **Skipped** | **6** |

| Question 9 | | |
| --- | --- | --- |
| **Are you aware of any other guidance or tools for patient engagement in PLS?** | | |
| Answer Choices | Responses | |
| No | 50.00% | 12 |
| Yes  Please tell us which one(s) | 50.00% | 12 |
|  | **Answered** | **24** |
|  | **Skipped** | **6** |

| Respondents | Yes  Please tell us which one(s) |
| --- | --- |
| 1 | More specifics available in the iEnvision pharma guide on PLSPs. |
| 2 | Based on the people and groups you have involved, I'd be surprised if you were not already aware of tools I know of...having said that, supporting tools and visuals are available from EUPATI and especially CTTI on the steps in the overall research process where patient engagement should take place (the respective documents or activities they inform), as well as, tools that help understand and prioritize patient engagement activities (by type and expected impact).   Also, I'd include the PEMAT for audio visual as well as the CDC Clear Communication index (which has a 'social media' version as well), since these other modes of communication are mentioned for PLS dissemination |
| 3 | Toolkit from Envision pharma |
| 4 | There are several for plain language summaries in general but very limited for plain language summaries of publications (PLSPs) which you are describing in this guidance. I would suggest changing the acronym in your guidance to PLSPs to be more clear of the distinction. In my experience, PLS are summaries of trial results specifically. |
| 5 | PFMD & Envision toolbox Open Pharma recommendations ISMPP PLS working group GPP4 under development |
| 6 | Recent EMWA article on patient publication steering committee with useful tips for medical writers |
| 7 | Envision Toolkit |
| 8 | Envision the Patient PLS Toolkit |
| 9 | UK national PPI standards +++ |
| 10 | Envision Toolkit, ISMPP workstream, Guidance from publishers (e.g. Adis/ Taylor&Francis), Open Pharma are general guidance on PLS. Not all specify the role of patients explicitly as PLS aren't just aimed at patients. |
| 11 | Envision toolkit ISMPP PLS perspectives working group |
| 12 | The Dutch government has recommendations for institutions and hospital app builders. https://www.w3.org/Translations/WCAG20-nl/. It contains also some recommendations for PLS. |

| Question 10 | | |
| --- | --- | --- |
| **Do you already use any guidance or tools in your patient engagement work in PLS (e.g. internal guidances, or others)?** | | |
| Answer Choices | Responses | |
| No | 33.33% | 8 |
| Yes Please specify which one(s) | 66.67% | 16 |
|  | **Answered** | **24** |
|  | **Skipped** | **6** |
|  |  |  |

| Respondents | Yes Please specify which one(s) |
| --- | --- |
| 1 | Specifically for publications we developed our own guidance |
| 2 | Other than internal CISCRP guidance, you likely know and use the same ones! |
| 3 | Internal guidance developed internally. |
| 4 | We have established internal processes and guidances to develop Lay Person Summaries (LPS) for all our trials; it is a commitment we have made in accordance with European regulations. Increasingly, we're pushing early development teams to also develop LPS based on an established template and process. |
| 5 | CISCRP |
| 6 | internal Novartis guidance based on various external recommendations |
| 7 | Envision toolbox |
| 8 | Internal guidance and patient engagement specialists within our business. |
| 9 | Internal guidelines |
| 10 | Envision Toolkit |
| 11 | Envision the Patient PLS Toolkit Client SoPs |
| 12 | As above plus experience |
| 13 | See above and internal guidance |
| 14 | Internal guidance |
| 15 | See above. I promised in my PHD-Proposal to make a PLS of every publication. |
| 16 | Lorem Ipsum |

| Question 11 | | |
| --- | --- | --- |
| **Is the 'interactive version' useful for you?** | | |
| Answer Choices | Responses | |
| Yes | 58.33% | 14 |
| No | 0.00% | 0 |
| If No, please specify why not | 41.67% | 10 |
|  | **Answered** | **24** |
|  | **Skipped** | **6** |

| Question 12 | | | | |  |  |
| --- | --- | --- | --- | --- | --- | --- |
| **Please read the following statements and choose the option that represents best your opinion** | |  |  |  |  |  |
|  | Strongly disagree | Disagree | Neutral | Agree | Strongly agree | Total |
| It is easy to interact with the content | 0.00% | 12.50% | 20.83% | 54.17% | 12.50% | 24 |
| The content is easy to read and understand | 0.00% | 4.17% | 12.50% | 58.33% | 25.00% | 24 |
| The design is appealing | 0.00% | 0.00% | 33.33% | 45.83% | 20.83% | 24 |
| I can easily find what I’m looking for | 0.00% | 12.50% | 41.67% | 33.33% | 12.50% | 24 |
| It is easy to navigate through it | 0.00% | 20.83% | 25.00% | 41.67% | 12.50% | 24 |
| Please briefly explain your choice (optional) |  |  |  |  |  | 2 |
|  |  |  |  |  | **Answered** | **24** |
|  |  |  |  |  | **Skipped** | **6** |

| Respondents | Please briefly explain your choice (optional) |
| --- | --- |
| 1 | Some of the expandable coloured bars would benefit from a 'click here' icon to make it clear they have an action/additional content (e.g. in the 'Step 2: Identify your target audience' section) |
| 2 | As above |

| Question 13 | |
| --- | --- |
| **Would you like to share any other considerations or feedback regarding this work?** | |
| **Answered** | **19** |
| **Skipped** | **11** |

| Respondents | Responses |
| --- | --- |
| 1 | It is a good overview |
| 2 | 1. Mention of cultural environment – there needs to be a tremendously greater focus on this point. Fully explain what cultural considerations are relevant, and how to understand and address them, including patient engagement activities specifically focused on just that (either as part of PLS activity or more broad organization initiatives the insights of which can then be applied for PLS). Also provide links to useful resources and mention there are professional resources that specialize in understanding and helping to address cultural aspects of health communications.   2. “suffering” – typically avoid this term and similar language when preferring to patient. “patients / people with disabilities” is all that need be said.  3. “layperson” – another ‘trigger’ word. I understand the need to use it (once) and define it. Thereafter, you shouldn’t. It can be demining to some, and therefore shouldn’t be used expect to acknowledge its existence and define.   4. Re: plan language writing, health literacy principles, and design skills: “may require training” is a tremendous understatement. There is an entire academic and professional discipline dedicated to researching, applying and further developing this expertise. I’m sure some of your workgroup members will agree on this point, as well as extending to the practice of patient-centricity more generally. Point is – it should mention (more than once) that there are professionals who specialize in this work and there absolutely needs to be training for some of the key stakeholders doing the work. Those resources should either be specifically mentioned or broadly referenced (including the availability of higher-ed programs).    5. Similar to points above, there could be a bit more on patient engagement work to understand and address issues facing target audience. Specifically, need to encourage folks to identify and leverage intra- or -extra-organizational initiatives that have or will gain these population specific insights. |
| 3 | Great work, thanks to the participants of the initiative. |
| 4 | N/A |
| 5 | -Overall, I thought this guidance was great. -Providing more information on how best to utilize target audience reviewers would be helpful (not just that they are important). -Would suggest changing to PLSP -43 pages sees a bit long -I am not sure creating the PLSP in parallel with the original article is realistic - in particular if you are writing a full PLSP (not just abstract length summary). -Information about how PLSPs qualify as secondary publication according to ICMJE would probably be useful  -Suggest adding recently released EMA glossary to the list |
| 6 | well done! |
| 7 | What is meant by "cultural environment" (p.11) Needs further explanation.  On p.12 please do not use the word "sufferers" it is a highly controversial term and should be avoided. Please instead use "Consider people who are living with disabilities" (Under "Format Considerations")  I think the wording in places about how it is "desirable" to have patient/lay input into PLS development should be amended - they SHOULD be involved. We need to get away from perpetuating the idea of patient involvement and co-creation being a "nice to have" rather than an essential part of the process (!) |
| 8 | The content is nicely organized, appealing and easy to digest and understand. |
| 9 | It would be useful to clarify when using PLS for HCP targets and when not. In principles, I would say HCPs (nurses, pharmacists) doesn't need plain language information as they are trained to understand scientific / medical language but to do evidence-based research. In some cases (e.g. if the paper/ information is targeting patient through HCPs), maybe a PLS "translation" is needed. Clarifying this in the guide would be useful. |
| 10 | The report is very long so it would help if some of the content could be reduced (such as the executive summary).  Suggest rewording the language in the guide and interactive tool (people who are suffering with disabilities) as this is quite negative. |
| 11 | Note - the 'find out more' page would not load for this guide: How-To Guide for Patient Engagement in the Early Discovery and Preclinical phases |
| 12 | well done! |
| 13 | Very impressive. This will really WORK. |
| 14 | Lots of involvement but were patients paid for their time?? |
| 15 | A very important step for patient engagement |
| 16 | There was one phrase that needed tweaking. Step 3 'Consider people that are suffering from disabilities (audio instead of reading a document)'  Use of 'suffering' is a no-no |
| 17 | 1. On page 12, as a disabled person I was shocked to read the phrase "suffering from disabilities". Whilst it may seem to an able-bodied person that physical disabilities causing pain and discomfort may cause suffering, the types of disabilities that have PLS access needs are in fact sensory or learning disabilities such as deafness, blindness, or autism. The disabled community and in particular these sub-groups of disabilities have a strong and well-established sense of community and pride and it is unbelievably offensive to suggest that because their sensory experiences are outside of able-bodied people consider "normal" , it must therefore cause suffering. What actually causes suffering is non-accessible and exclusionary atmospheres, such as the one created when reading this sentence. Please change it immediately. |
| 18 | I support this and we provide PLS for many of our publications. However doing this through formal co-creation is expensive and needs funds we don't have. There is a danger that patients push for co-creation so hard that people decide not to engage at all. |
| 19 | Just a compliment |

| Question 14 | | |
| --- | --- | --- |
| **I would like to keep in touch** |  |  |
| Answer Choices | Responses | |
| I would like to receive updates | 86.67% | 13 |
| I'm interested to pilot this work | 53.33% | 8 |
| Add here your name and email |  | 16 |
|  | **Answered** | **15** |
|  | **Skipped** | **15** |
