# Supplementary material for: A practical ‘How-To’ Guide to plain language summaries (PLS) of peer-reviewed scientific publications: results of a multi-stakeholder initiative utilizing co-creation methodology
Source: Res Involv Engagem. 2022 Jun 2;8:23. doi: 10.1186/s40900-022-00358-6 (PMC9164486; doi:10.1186/s40900-022-00358-6)
Supplement: Supplementary file 5 — Additional file 5: ‘How-To’ Guide: executive summary and annexes. [file 40900_2022_358_MOESM5_ESM.pdf]

How-To Guides for Patient Engagement

PATIENT FOCUSED MEDICINES DEVELOPMENT

Plain language summaries (PLS) of peer-reviewed publications and conference presentations: practical 'How-To' Guide for multi-stakeholder co-creation

This How-To guide is part of a series of PFMD How-To guides that have been co-created in a multi-stakeholder environment built with the Patient Engagement Quality Guidance as a starting point. All How-To's are connected and provide a full set of instructions on how to involve patients across the research, development, and delivery of medicines.

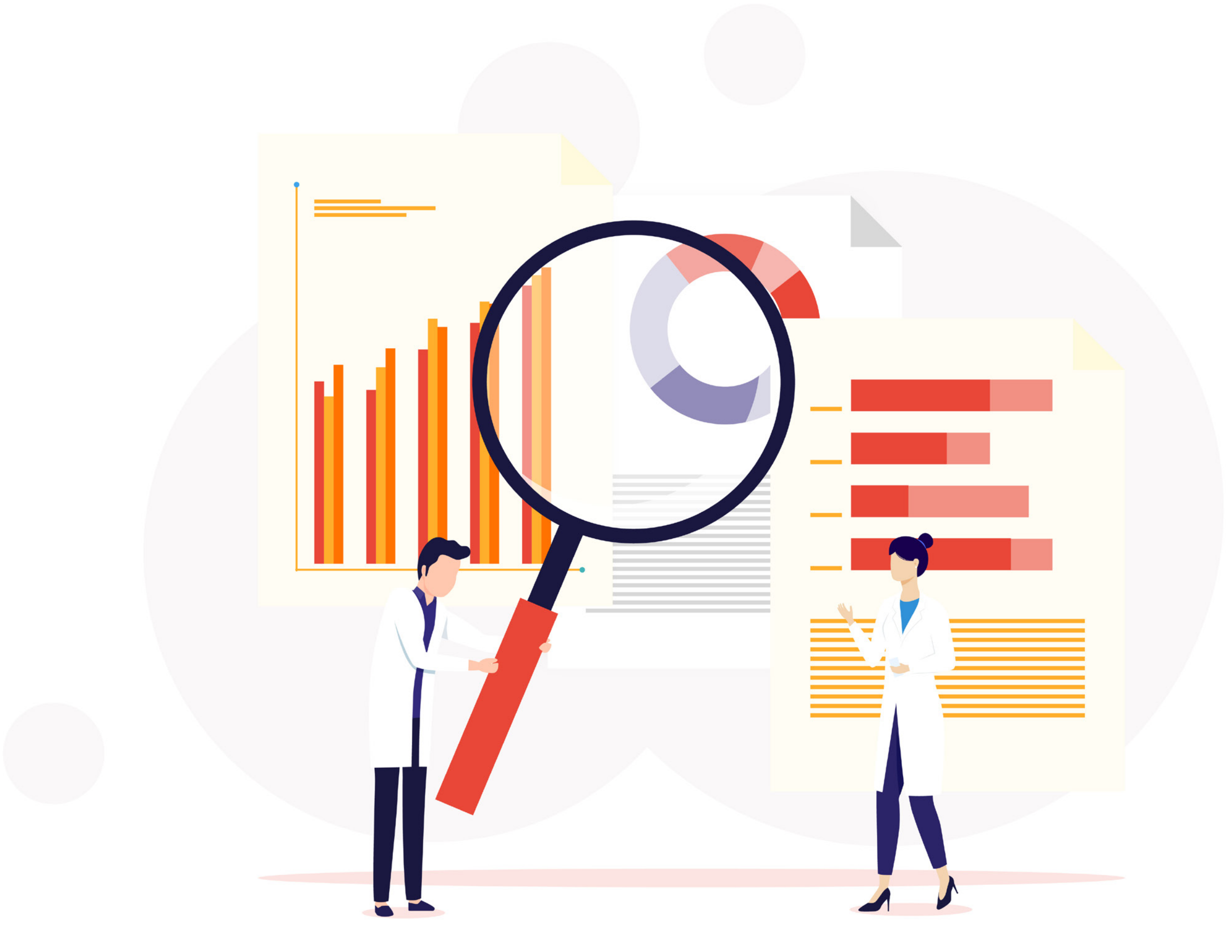

The guides are part of the **Patient Engagement Management Suite** - a hub for effective and practical patient engagement.

Executive Summary

This How-To guide is part of a series from Patient Focused Medicines Development (PFMD) that provides a set of instructions on how to involve patients in the co-creation of plain language summaries (PLS) of peer-reviewed publications, including journal articles and conference presentations.

Section 1 - Introduction and Overview of the work

- Peer-reviewed publications and conference presentations are a means for medical researchers to present their research to their peers, for example, the results of a clinical trial.
- PLS tailor this information so it is suitable for a broader audience in terms of readability and understanding, and are valuable to a broad range of readers, including patients, patient organisations, caregivers, and the general public.
- PLS are also valuable to healthcare professionals (HCPs) and non-specialist physicians to help generate dialogue and more focused communication with their patients.
- Patient involvement and engagement in the development of a publication related PLS is often restricted to the later stages of their creation (e.g. the review process).
- The aim of this document is to:
  - Provide a How-To guide for the creation and dissemination of of a peer- reviewed journal publication or congress presentation
  - Strongly recommend co-creation with patients at every stage of development of the PLS
- The How-To guide has been co-created with a wide variety of stakeholders, including: patient representatives, industry members, publishers, researchers, medical communication agencies and public officials involved in research bodies.
- The How-To guide is organised into a seven-step approach, and considers the seven quality criteria of the Patient Engagement Quality Guidance (PEQG) in the first four steps. The guide has been developed for anyone who needs to write or contribute to a PLS of a peer-reviewed journal publication or congress presentation.

Section 2 - Ethical considerations for PLS

- PFMD seeks to foster the involvement of patients in all stages of the medicines life cycle. Given that patients can only fully participate if they are well-informed about current medical developments, PLS of scientific publications and of contributions to legitimate scientific congresses are at the core of this.
- While there is a clear need for scientific results to be shared with the public, it is important this is done in an ethical and responsible way, to avoid the potential for misinterpretation and a negative impact on patient care.
  As such, this How-To guide has identified some key ethical principles that should act as a guide throughout the creation of a publication related PLS.

Section 3 - Step-wise approach for PLS co-creation

Step 1: Rationale and Scope of your PLS

- The justification for the creation of a PLS should be considered (to demonstrate absence of promotional motivation), along with plans for where the PLS will be published, and the resources that will be required for its co-creation.

Step 2: Identify your target audience

- Knowing the target audience for the PLS is vital to determine what their needs are, and therefore what information it should contain.

Step 3: Consider dissemination channels for PLS.

- Dissemination of the PLS will also affect content and format, so should be considered early on. Certain channels (such as social media platforms) might work better for certain audiences in terms of reach and engagement.
- PLS will be submitted for publication at the same time than the publication in a peer-reviewed journal and therefore the journal requirements should also be considered before writing begins. This also applies to PLS of conference presentations, where the conference may have content and formatting requirements that must be complied with.

Step 4: Identify your key stakeholders for co-creation of PLS.

- Consider which of the key stakeholders from your target audience (identified in Step 2 - Identify your target audience) to bring into PLS co-creation. This will help ensure that the PLS is understandable and relevant for each target audience.
- There are various activities in the creation of a PLS that can incorporate co-creation, such as selecting publications/presentations for which to develop a PLS; planning the content writing and reviewing the PLS.
- Provided they have some of the relevant skills required, patients should be participants throughout the creation of a PLS.

Step 5: Write your PLS.

- The PLS should be written with the target audience in mind, to ensure factors like the reading age, type of visual and audio formats, and the type of language you use (based on literacy level) are appropriate.
- Visuals and infographics can help engage the reader, and it is important to think about where these can be used most effectively.
- Readability and suitability tools are available to help check how easy your publication PLS is to read and understand. It is also helpful to test the PLS with someone from your target audience and ask them to provide feedback.

Step 6: Disseminate your PLS.

- Once published, the PLS could be disseminated by a variety of sources including the journal that published it, the institutions of the authors, the authors themselves, patients, advocacy groups, medical societies and HCPs.
- The PLS could be shared via social media, print copies, repositories such as PubMed, and other relevant websites.
- However the PLS is shared, it should always reference the scientific manuscript or contain links to the scientific source.

Step 7: Track dissemination and measure success.

- It is helpful to establish a strategy for monitoring the impact of any PLS, and this is an important way to empower the entire process and systematize the assessment of its value and impact.
- Depending on where a PLS is hosted, various metrics will be available to you to track how it is being used.

Section 4 - PLS of Conference presentations

Although focused on PLS of peer-reviewed journal publications, much of the guide also applies to PLS of conference presentations. However, there are a few additional considerations such as: extent of patient attendance, other delegates at the conference, PLS for abstract submission, PLS data content, data presentation, posters, conference requirements, metrics to track dissemination, dissemination strategy beyond the conference itself.

Section 5 - Considerations for quality PE (Patient Engagement)

This How-to Guide (as all PFMD How-to Guides) has been built to be used alongside the PFMD Patient Engagement Quality Guidance (PEQG) which defines seven Quality Criteria for good patient engagement. These criteria have been adapted as practical considerations and utilised where they fit best.

Section 6 - Acknowledgements

Section 7 - Annexes (Annex 1 Glossary; Annex 2 Resources for Writing your PLS; Annex 3 Examples of PLS of peer-reviewed publications and where to find them; Annex 4 PE Quality Guidance definitions)

To supplement this How-To guide, a number of resources have also been compiled to assist in the creation of PLS, including plain language resources, useful videos and toolkits, and examples for published PLS.

Annex 1
Glossary
Additional resources

It is important to consider that the general population is not accustomed to the scientific jargon and terminology commonly found in peer-reviewed research articles. As such, when writing up the PLS, the author needs to be faithful to the research presented, but will undoubtedly also use a minimum amount of technical terminology.

It is important to only include terms patients and the public may hear from medical clinics, and any scientific terms should be explained in the PLS. To help the reader better understand and navigate through the PLS, a glossary is also highly recommended.

A glossary may include scientific terminology, acronyms and other related jargon present in the PLS. Some examples include:

- a simplified definition of a term
  - eg. arrhythmia: irregular heartbeat
- a synonym
  - eg. abdomen: belly, stomach
- acronyms
  - eg. ICU: Intensive Care Unit

Glossary formatting varies from a simple list of alphabetical terms to a table-like structure, and could be suggested by the publisher.

The most important aspect is that the information provided is clear and easy to quickly pick up. The glossary should also be reviewed by the patient advocates and lay readers involved in reviewing the PLS to ensure that all necessary terms are present, as well as potentially suggesting changes or additional terms. These would then be forwarded to the author for consideration.

1. Resources for Lay Terminology Glossaries

Below are resources that can be helpful when looking for lay terms to use in a plain language summary.

- The European Patients' Academy Glossary
  https://www.eupati.eu/glossary/
  Searchable online glossary covering key terms used in medicines development
- National Institute for Health Research (NIHR) INVOLVE Jargon Buster
  https://www.invo.org.uk/resource-centre/jargon-buster/
  Searchable online glossary provides plain language definitions of terms used in public involvement in research
- National Cancer Institute (NCI) Dictionary of Cancer Terms
  https://www.cancer.gov/publications/dictionaries/cancer-terms
  Searchable online glossary defining terms related to cancer and medicine

- Health Technology Assessment International (HTAi) HTAi Consumer and Patient Glossary
  https://htai.org/wp-content/uploads/2018/02/PCISG-Resource-ENGLISH-PatientandConsumerGlossary-Oct09.pdf
  Provides plain language terms for words used in health technology assessment documents
- The plain language Glossary of Evaluation Terms for Informed Treatment choices (GET-IT)
  https://getitglossary.org/
  Searchable online glossary provides plain language definitions of health research terms
- http://www.plainenglish.co.uk/files/alternative.pdf
  Provides plain language alternatives to frequently used complex words and phrases, plus a list of 'words and phrases to avoid'
- Centers for Disease Control and Prevention (CDC) – Everyday Words for Public Health Communication
  https://www.cdc.gov/other/pdf/everydaywordsforpublichealthcommunication.pdf
  Glossary of plain language alternatives for health terms, using real examples of technical sentences rewritten in plain language
- Stanford University Definitions & Lay Glossary of Medical Terms:
  https://researchcompliance.stanford.edu/panels/hs/forms/definitions
- University of Iowa Medical Terms in Lay Language:
  https://hso.research.uiowa.edu/medical-terms-lay-language
- University of Florida Glossary of Lay Terms for Use in Informed Consent Forms:
  http://irb.ufl.edu/irb01/forms/glossary.html
- University of California Davis Glossary of Lay Terminology:
  https://research.ucdavis.edu/wp-content/uploads/Glossary-of-Lay-Terminology_12-15-14.pdf
- Loma Linda University Glossary of Lay Terms:
  https://researchaffairs.llu.edu/responsible-research/human-studies/resources-for-human-studies/handbook-for-human-research-protections-program/glossary-of-lay-terms
- McLaren Health Care Corporation Human Research Protections Program Glossary of Terms:
  http://www.mclaren.org/uploads/Public/Documents/Corporate/lay_language_1.pdf
- University of Kentucky Research Glossary of Lay Terms:
  https://www.research.uky.edu/uploads/ori-d780000-layterm-pdf
- VA Portland Health Care System Glossary of Lay Terms for Use in Preparing Consent Forms:
  https://www.portland.va.gov/research/documents/hrpp/glossary-of-lay-terms.pdf
- SingHealth DukeNUS Academic Medical Centre Glossary of Lay Terms for use in Consent Documents:
  https://www.singhealthdukenus.com.sg/research/rice/Documents/Glossary%20of%20lay%20terms%20[2016-07-26].pdf
- Lebanese American University Medical to Lay Terminology:
  https://gsr.lau.edu.lb/irb/forms/medical_lay_terms.pdf
- Think local act personal Jargon Buster:
  https://www.thinklocalactpersonal.org.uk/Browse/Informationandadvice/CareandSupportJargonBuster/
- National Institute for Health Research INVOLVE Jargon Buster:
  https://www.invo.org.uk/resource-centre/jargon-buster/
- EMA medical terms simplifier
  https://www.ema.europa.eu/en/documents/other/ema-medical-terms-simplifier_en.pdf
  Plain-language description of medical terms related to medicines use

2. Glossary of terms

Below are definitions of key terms that are used throughout the document. The Glossary is built on the definitions reported in several guidances[6].

Analysis plan (also known as Statistical analysis plan): A document that contains a more technical and detailed elaboration of the principal features of the analysis described in the protocol, and includes detailed procedures for executing the statistical analysis of the primary and secondary variables and other data (Source: FDA, 1998).

Carer/caregiver: A person who helps a patient with daily activities, health care, or any other activities that the patient is unable to perform himself/herself due to illness or disability, and who understands the patient's health-related needs. This person may or may not have decision-making authority for the patient and is not the patient's healthcare provider (Source: FDA, 2018).

Congress: Regular coming together on a representational basis of several hundreds - or even thousands - of individuals belonging to a single professional, cultural, religious or other group. A congress is often convened to discuss a particular subject. Contributions to the presentation and discussion of the subject matter come only from members of the organising body (ICCA, 2020).

Conference: Participatory meeting designed for discussion, fact-finding, problem solving and consultation. As compared with a congress, a conference is normally smaller in scale and more select in character - features which tend to facilitate the exchange of information (ICCA, 2020).

Healthcare professionals (HCP): Refers to practitioners, including physicians, nurses, pharmacists, dentists, respiratory therapists, physical therapists, technologists, or any other practitioners or allied health professionals that have a role in using a device for human use (Source: FDA, 1).

Health literacy: The degree to which individuals have the capacity to obtain, process, and understand basic health information and services needed to make appropriate health decisions. Health literacy also includes numeracy skills—such as calculating cholesterol and blood sugar levels, measuring

[6] USA. Food and Drug Administration (FDA). (1998) E9 Statistical Principles for Clinical Trials, Federal Register Volume 63, Number 179. Available at: https://www.govinfo.gov/content/pkg/FR-1998-09-16/html/98-24754.htm

USA. Food and Drug Administration. (2018) Patient-Focused Drug Development Glossary. Available at: https://www.fda.gov/drugs/development-approval-process-drugs/patient-focused-drug-development-glossary

Global. International Congress and Convention Association (ICCA). (2020) Frequently asked questions. Available at: https://www.iccaworld.org/aeps/aeitem.cfm?aeid=909#:~:text=A%20congress%20is%20often%20convened,be%20either%20multiannual%20or%20annual

USA. Food and Drug Administration. (2019) Code of Federal Regulations, Title 21, Chapter I, Subchapter H, Part 810. Available at: https://www.accessdata.fda.gov/scripts/cdrh/cfdocs/cfcfr/cfrsearch.cfm?fr=810.2

Global. Wiley. [Internet] Open Access home page. Available at: https://authorservices.wiley.com/open-research/open-access/index.html

EU. European Patients Forum (EPF).(2020) What is a Patient Organisation? Available at: https://www.eu-patient.eu/Members/what-is-a-patient-organisation/

USA. National Institutes of Health, National Cancer Institute. [Internet] NCI Dictionary of Cancer Terms. Available at: https://www.cancer.gov/publications/dictionaries/cancer-terms/def/peer-reviewed-scientific-journal

Global. Patient focused medicines development (PMD). [Internet] Available at: https://patientfocusedmedicine.org/

EU. European Medicines Agency. (2017) Summaries of Clinical Trial Results for Laypersons. Available at: https://ec.europa.eu/health/sites/health/files/files/eudralex/vol-10/2017_01_26_summaries_of_ct_results_for_laypersons.pdf

EU. European Medicines Agency. (2010) EU Clinical Trials Register Glossary. Available at: https://www.clinicaltrialsregister.eu/doc/EU_Clinical_Trials_Register_Glossary.pdf

USA. Food and Drug Administration. (2019) Patient-Focused Drug Development: Methods to Identify What Is Important to Patients Guidance for Industry, Food and Drug Administration Staff, and Other Stakeholders. Available at: https://www.fda.gov/media/131230/download

medication doses, and understanding nutrition labels—and knowledge of health topics (Source: FDA, 2018).

Open access (OA): A publication that is freely available to read, download, and share (Source: Wiley, internet).

Patient organisation: Not-for profit organisations which are patient focused, and whereby patients and/or carers (the latter when patients are unable to represent themselves) represent a majority of members in governing bodies (Source: EPF, 2017).

Peer-reviewed publication: A publication written by scientists and evaluated for technical and scientific quality and correctness by other experts in the same field (Source: NIH, Internet).

PFMD (Patient focused medicines development): An open, independent global coalition of health stakeholders with aims to transform the way in which we understand, engage, and partner with patients globally in the design and development of research and medicines by focusing on unmet patient needs (Source: PFMD, 2020).

Plain language summary (PLS): A non-technical summary of clinical trial results or other content in a journal article or congress presentation. This How-To guide refers to PLS for publications and conferences and do not include PLSs that are created per EU regulations (ie, layperson summaries) that follow a certain regulatory rigor (Reference: EMA, 2017).

Primary endpoint(s): The main result that is measured at the end of a study to see if a given treatment worked (e.g., the number of deaths or the difference in survival between the treatment group and the control group). What the primary endpoint will be is decided before the study begins (Source: EMA, 2010).

Protocol (also known as Study protocol): A document that describes the objective(s), design, methodology, statistical considerations and organisation of a trial. The term protocol refers to the protocol, successive versions of the protocol and protocol amendments (Source: EMA, 2010).

Secondary endpoint(s): Results that are measured at the end of a study, in addition to the main result (primary endpoint) to see if a given treatment worked. Secondary endpoints can explore other aspects of the treatment (Source: EMA, 2010).

Annex 2

Resources for Writing your PLS

Additional resources

1. Guides to health literacy and plain language writing

Below are resources that can be helpful when looking for lay terms to use in a plain language summary.

- US CDC plain language resources
  https://www.cdc.gov/healthliteracy/developmaterials/plainlanguage.html
- The Multi-Regional Clinical Trials Center Health Literacy in Clinical Research Toolkit
  https://mrctcenter.org/health-literacy/tools/overview/
  Collection of tools aimed at creating clear communication materials for clinical trials. 'Best practices' section contains useful resources on plain language, numeracy, design and usability testing.
- How to write in plain English – Plain English Campaign
  http://www.plainenglish.co.uk/files/howto.pdf
  General guide to the principles of plain language writing, including a summary key points and a list of alternatives to 'words to avoid'
- How to write medical information in plain English – Plain English Campaign
  http://www.plainenglish.co.uk/files/medicalguide.pdf
  Gives 'before' and 'after' examples of plain-language medical writing, plus a glossary of medical terms
- Centers for Disease Control and Prevention (CDC) – Everyday Words for Public Health Communication
  https://www.cdc.gov/other/pdf/everydaywordsforpublichealthcommunication.pdf
- Consider Patient Information Forum (PIF) resources, especially communicating numbers and risk.
  https://pifonline.org.uk/resources/
- Plain Language Summaries of Publications Toolkit – Envision Pharma Group/PFMD
  https://www.envisionthepatient.com/plstoolkit/
  An evidence-based, freely-available resource specific to support development of PLS of publications, including a template, QC checklist for writers, cover sheets for patient and sponsor reviewers, and a guide summarising helpful plain-language glossaries
- Slides from Jan Seal-Roberts (Publishing Director, Adis | Springer Healthcare) and Sarah Griffiths (Communications Team Leader, Oxford PharmaGenesis) ISMPP Annual Meeting 2020 workshop
  https://synapse.pfmd.org/resources/patient-lay-summaries-plain-language-summaries-understanding-the-differences-and-how-best-to-use-these-options
  An all-in-one overview of the nuances around the Why? What? How? Who? When? on publication plain language summaries, including distinction versus trial results summaries
- Standards for the reporting of plain language summaries in new Cochrane Intervention Reviews 2013 – Cochrane Methods
  https://methods.cochrane.org/sites/default/files/public/uploads/pleacs_2019.pdf
  A summary of quality standards for PLS of Cochrane systematic reviews, including guidance on content and structure to help writers meet each standard

- Writing and testing plain language – Model Systems Knowledge Translation Center
https://msktc.org/lib/docs/KT_Toolkit/MSKTC_Plain_Lang_Tool_508.pdf
A tool that presents the principles of plain language writing and how to test the reading level of your writing. Includes 'before and after' examples and a glossary of simpler word choices
- Universal Patient Language Guidance
https://www.upl.org/
- Examples of Active and Passive Voice
https://examples.yourdictionary.com/examples-of-active-and-passive-voice.html
- Establishing a patient publication steering committee: A case study with insights for medical writers
https://journal.emwa.org/writing-for-patients/establishing-a-patient-publication-steering-committee-a-case-study-with-insights-for-medical-writers/
Summarise how UCB Pharma, a global biopharmaceutical company, plans to partner with patients to establish a Patient Publication Steering Committee (PPSC) and share insights on how medical writers could support PPSCs

2. Maximize white space

- This is a link to the science around white space
https://www.interaction-design.org/literature/article/the-power-of-white-space. An offer for a link to the use of white space in UX will come up as well.
- Another link more simply describes the value of white space
https://novacreative.com/5-benefits-of-using-white-space-in-design/
- An example that could be shown here is from the CDC
https://health.gov/healthliteracyonline/display/section-3-4/

3. Useful videos

- MedComms networking webinar – Patient involvement in scientific communications
https://networkpharma.tv/2019/11/20/patient-involvement-in-scientific-communications/
Dr Lauri Arnstein (Envision Pharma Group) provides an overview of patient involvement in medical publications, including PLS of publications and patient authorship
- MedComms networking webinar – Plain language summaries: what are they, and why should we consider including these in our publications?
https://networkpharma.tv/2019/05/29/plain-language-summaries-what-are-they-and-why-should-we-consider-including-these-in-our-publications/
Jan Seal-Roberts (Adis Journals) gives the publisher's perspective, discussing what PLS are and the opportunities they can offer
- MedComms networking webinar – Adapting your writing for patients and the public
https://networkpharma.tv/2019/10/16/adapting-your-writing-for-patients-and-the-public/
Hannah Bridges (HB Health Comms LTD) presents the principles of clear, effective plain language communications
- International Society for Medical Publication Professionals University – The rapid adoption of plain language summaries in medical communications: Hope or hype?
https://ismpp.memberclicks.net/ismpp-u---november-2019?servId=10046
Only available for ISMPP members

4. Examples of readability tools

- Readable
  readable.com
  This paid tool tests readability, spelling and grammar and highlights text to show where potential issues are. It scores the text from A (most readable)–E (least readable) and provides grade levels from other commonly used readability tools
- Hemingway Editor
  http://www.hemingwayapp.com/
  This paid tool highlights text to show long sentences and other complex areas. It provides a grade score for readability
- Clear Communication Index User Guide
  https://www.cdc.gov/ccindex/tool/index.html
  The Clear Communication Index (Index) provides a set of research-based criteria to develop and assess public communication products.

The following tools are available at
https://readabilityformulas.com/free-readability-formula-tests.php

- **Flesch Reading Ease**
  Checks average sentence and word length. Gives a score from 0–100 (higher score = text is easier to read). Ideal score is between 70 to 80 (equivalent to school grade level 8).
- **Flesch-Kincaid**
  Translates the 0–100 from the previous tool score to a grade reading level. Ideal score is 7 or 8, scores of 12 or more are too difficult for most people to understand
- **Gunning Fog**
  Checks average sentence length and number of words of 3 or more syllables. Ideal score is 7 or 8, scores of 12 or more are too difficult for most people to understand
- **SMOG Index**
  Measures number of sentences and words of 3 or more syllables. Gives a grade reading level

5. Suitability Assessment tools

The following suitability tools are designed for assessing health information:

- **Patient Education Materials Assessment Tool for Printable Materials (PEMAT-P)**
  https://www.ahrq.gov/ncepcr/tools/self-mgmt/pemat-p.html
  Gives separate scores for understandability and whether the reader can take the correct action after reading (actionability). The actionability score may or may not be relevant for your PLS. Can be downloaded as an Excel file that will calculate the scores for you.
- **Suitability Assessment of Materials (SAM)**
  http://aspiruslibrary.org/literacy/SAM.pdf
  Focuses on understandability, and includes scores for visuals, layout and cultural considerations.

Annex 3

Examples of PLS and where to find them

Additional resources

This section provides some recent examples of PLS of publications. PLS are currently very diverse in terms of their content, layout and how they are published by journals. These examples should give you an idea of this range. The list is not exhaustive, but might help you define more easily which type of PLS would best fit your needs.

Text-only PLS, published within the main article

- https://rd.springer.com/content/pdf/10.1007%2Fs40744-017-0080-4.pdf
  This short text-only PLS sits below the main abstract. Note that the authors have used effective layout principles such as bolded text, question headers and numbered lists.
- https://www.dovepress.com/relationship-between-pain-reduction-and-improvement-in-health-related--peer-reviewed-fulltext-article-JPR
  This short text-only PLS sits below the main abstract. Note that the authors have split the content out into bullets, rather than using plain text.

Text-only PLS, published as a supplement to the main article

- https://adisjournals.figshare.com/articles/A_Randomized_Double-Blind_Efficacy_and_Safety_Study_of_PF_05280586_a_Rituximab_Biosimilar_Compared_With_Rituximab_Reference_Product_MabThera_in_Subjects_With_Previously_Untreated_CD20-Positive_Low-Tumor-Burden_Follicular_Lymphoma_LTB-FL_/10282727
  This longer text-only PLS is hosted on Figshare, which means it can also be found as a standalone document through Figshare.

  The main article (https://link.springer.com/article/10.1007/s40259-019-00398-7) contains a reference to the PLS, which is listed as an enhanced digital feature.

Text and visuals PLS, published as a supplement to the main article

- https://static-content.springer.com/esm/art%3A10.1007%2Fs43441-020-00115-5/MediaObjects/43441_2020_115_MOESM1_ESM.pdf
  This colour text and visuals PLS is hosted by the journal as electronic supplementary material, via a link in the main article (https://link.springer.com/article/10.1007%2Fs43441-020-00115-5). Ideally, the main article would contain a reference to the PLS to make it easier to find.

Short text and visual PLS, published as a figure in the main article

- https://academic.oup.com/ofid/article/6/2/ofz007/5288627
  This short colour text and visuals PLS is Figure 3 in the manuscript. Ideally it would be the first figure of the manuscript to be more visible, and be saved on a sharing platform like Figshare for easier access. In that case, the PLS should contain a link to the main article, for readers who accessed the PLS first but then wanted more detail about the study.

Text and visuals PLS, published within the main article and as a supplement to the main article

- https://adisjournals.figshare.com/articles/The_Association_Between_Type_2_Diabetes_and_Cardiovascular_Disease_The_For_Your_SweetHeart_Survey/7546817
  This one-page text & visuals PLS is hosted in two places: on Figshare via a link in the main

article, and as Figure 1 in the main article (https://link.springer.com/article/10.1007/s12325-019-0871-9). Ideally this PLS would also contain a link that readers can follow back to the main article.

Text and visuals PLS, hosted by a patient group after manuscript publication

- https://www.acromegalycommunity.org/images/studies/AcroVoice_preference_study_PLS_9Jan19.pdf
  This text and visuals PLS is based on a journal article (https://www.ncbi.nlm.nih.gov/pmc/articles/PMC6373299/pdf/11102_2018_Article_933.pdf) and contains a link to the main article. It was not published by the journal but was instead hosted on a patient group website.

Annex 4
PE Quality Guidance definitions

The Patient Engagement Quality Guidance ([PEQG](https://pemsuite.org/peqg/)[5]) was co-created to help all stakeholders set up partnerships and projects collaboratively. It can be used:

- when having first discussions with new partners to identify and align on shared purpose for the project, roles and responsibilities, accessibility considerations, feedback loop etc.;
- when assessing ongoing projects to see if there are aspects where you could improve to increase the level of engagement and participation;
- when retrospectively assessing completed projects to identify areas of improvement for future projects.

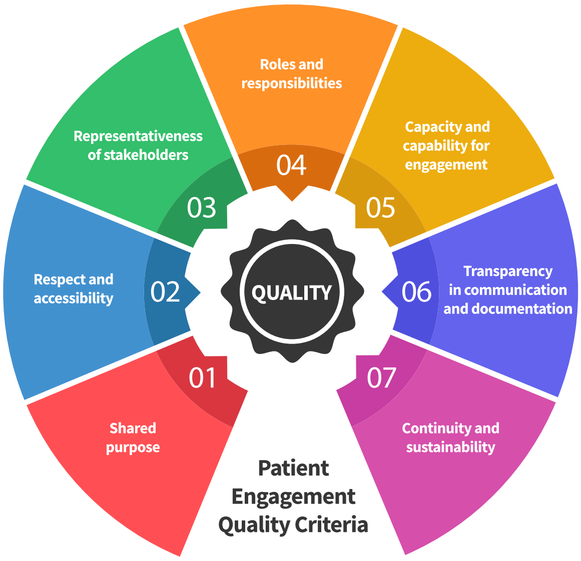

Shared Purpose

This refers to the project's aims and outcomes that all stakeholders taking part should agree on before starting the project. Consider putting in place processes to help facilitate discussions between all stakeholders to identify each other's values, expectations and objectives, and review and discuss priorities in the planning of the project.

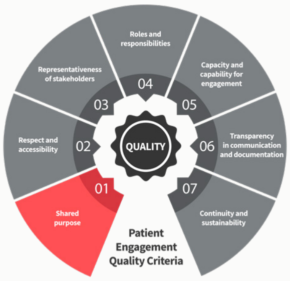

It can be valuable to enable stakeholders to exchange views openly to understand the scope and objectives of the project, acknowledging that some of their objectives may differ. All parties concerned should also have a shared written description of the common goals of the project.

Respect and accessibility

This refers to (1) respecting each other, and respectful interactions within the project to be established among partners, and (2) openness to and inclusion of individuals and communities (to the project) without discrimination. Considerations to ensure good conditions to implement the project should be made from the beginning.

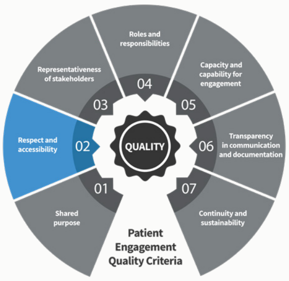

[5] https://pemsuite.org/peqg/

For example:

- simplification of wording
- budget and payment considerations
- cultural adaptations to procedures
- practicalities such as meeting timing, location and format
- accessibility of project materials
- written co-developed rules of conduct

Accessibility to participate may be facilitated by enabling multiple ways to involve stakeholders who could benefit from and/ or contribute to the project.

For example, patients with cognitive impairment might need more time to go through project material, or need printed versions rather than electronic documents or PDFs for easier reading.

Representativeness of stakeholders

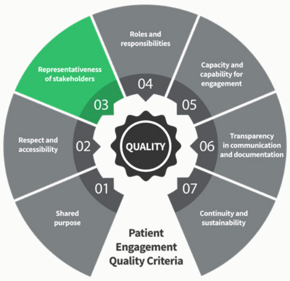

This refers to the mix of people you involve, which should reflect the needs of the project, and the interests of those who may benefit from project outputs (for example, target population).

Consider diversity in expertise, experience, demographics, and other relevant criteria for inclusion.

When selecting PE stakeholders, patients, attention will be given to awareness of the diversity required to achieve visible representative voice.

Roles and responsibilities

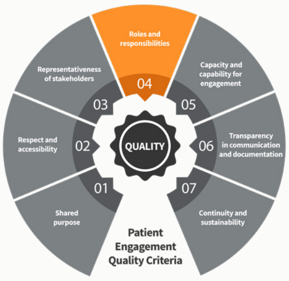

This refers to the need for clearly agreed, and ideally co-created roles and responsibilities, in writing, addressing that all aspects of project needs will be established upfront and revisited regularly

Capacity and capability for engagement

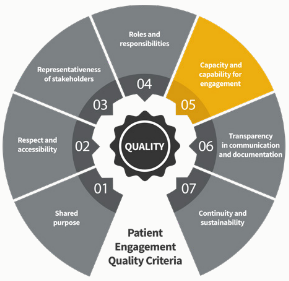

This refers to (1) capacity as having relevant and dedicated resources from all stakeholders (for example, providing a dedicated point of contact by the sponsor and having allocated sufficient time by all stakeholders to allow genuine engagement); and (2) capabilities for all stakeholders to enable meaningful engagement (for example, the level of knowledge, expertise and training stakeholders might need to deliver PE activities throughout the project).

Consider supporting stakeholders to build the required capacity and capabilities for this project in different forms of training both with sponsor organisations and with each stakeholder (for example, helping to understand the context, processes, relevant terminology etc.). Both capacity and capability building are intended to facilitate participation and lower barriers to collaborate. Stakeholders can be given access to learning resources and given dedicated support (if needed). Capability needs may vary depending on the project needs, but also e.g. personal circumstances of PE representatives.

Transparency in communication and documentation

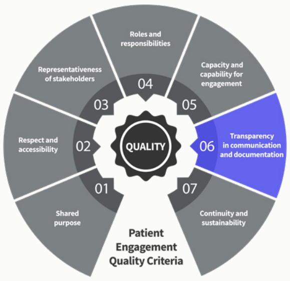

This refers to the establishment of communications plan and ongoing project documentation that can be shared with stakeholders. Communication among stakeholders must be open, honest and complete. In addition, adequate up-to-date documentation must facilitate communication with all stakeholders throughout the project. Consider proactively and openly sharing progress updates throughout the project externally. In addition, communicating outcomes of the project to all stakeholders and how their contribution was of value to the success of the project is critical.

Continuity and sustainability

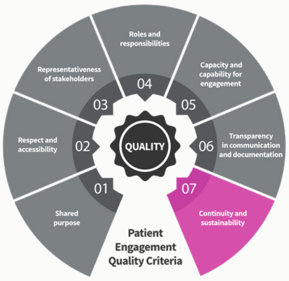

This refers to the smooth progression of the project, as well as efforts to maintain ongoing relationships with stakeholders. Consideration should be given for the role of stakeholders beyond a single project.

When starting the project, consider including in your project plan the actions needed for maintaining expected flow of the project from beginning to end. Create a plan to nurture relationships with your partners and stakeholders involved during the project, and when needed and requested, beyond the project as well. For all stakeholders successful planning and personal and organisational resilience should be anticipated.
